# The Synergistic Beneficial Effect of Thyme Honey and Olive Oil against Diabetes and Its Complications Induced by Alloxan in Wistar Rats

**DOI:** 10.1155/2021/9949056

**Published:** 2021-09-20

**Authors:** Hajar Lafraxo, Meryem Bakour, Hassan Laaroussi, Asmae El Ghouizi, Driss Ousaaid, Abderrazak Aboulghazi, Badiaa Lyoussi

**Affiliations:** Laboratory of Natural Substances, Pharmacology, Environment, Modeling, Health, and Quality of Life (SNAMOPEQ), Faculty of Sciences Dhar Mehraz, Sidi Mohamed Ben Abdellah University, Fez, Morocco

## Abstract

Diabetes is a metabolic disorder characterized by a chronic increase in blood glucose. Owing to the limitations observed with antidiabetics in modern medicine, medicinal plants and bee products are known as good matrices for the search for new antidiabetic molecules. The present study focused on the evaluation of the hypoglycemic and the protective properties of two natural products widely used in complementary and alternative medicine (thyme honey and olive oil). To achieve this, the study was carried out on Wistar rats rendered diabetic by the injection of a single dose of alloxan monohydrate (65 mg/kg body weight (BW)). First, the physicochemical characterization and the phytochemical analysis of thyme honey and olive oil were carried out, and then *in vivo* study was conducted on 42 Wistar rats divided into seven groups: three groups were normal, one group was untreated diabetic, and three groups were diabetic rats treated with thyme honey (2 g/kg BW) or olive oil (10 mL/kg BW) or their combination ((1 g/kg BW of thyme honey) and (5 mL/kg BW of olive oil)). During the experiment, the glycemia was measured regularly every 10 days. After 30 days of treatment, the rats were sacrificed. The serum and urine were analyzed to determine hepatic enzymes levels (AST, ALT, ALP, and LDH), lipidic profile (total cholesterol, triglycerides, high-density lipoprotein, low-density lipoprotein), and kidney parameters (urea, uric acid, creatinine, total protein, sodium, potassium, and chloride). The liver, pancreas, and kidneys were analyzed to evaluate their histological changes and to determine their enzymatic antioxidant content (catalase, GSH, and GPx) and the levels of MDA. The results obtained showed that thyme honey or olive oil, and especially their combination, improved significantly the blood glucose levels and they protect against metabolic changes and the complications induced by diabetes.

## 1. Introduction

Diabetes is a metabolic disease characterized by a chronic increase in blood glucose levels; it occurs when the pancreas does not produce enough insulin due to the autoimmune destruction of *ß* cells (type 1 diabetes) or when the body cells become resistant to insulin (type 2 diabetes) [1]. Around the world, the number of people with diabetes continues to increase at an alarming rate. According to Wild et al., the statistics in 2000 was 171 million and it is expected to increase to 366 million diabetes people in 2030 [2]. Diabetes places a heavy burden on diabetic people and their families, as well as on health systems and national economies, due to medical costs, the disease management, as well as the complications which are responsible for significant economic and human losses [3]. The therapeutic strategies of type 1 diabetes are based on the prevention against the destruction of beta cells by autoimmunity, maintaining immune homeostasis, and the restoration of glycometabolic control [4]. Currently, the standard treatment for type 1 diabetes is the lifelong administration of exogenous insulin [5]. The problem that arises is the difficulty to achieve daily blood sugar control, which ultimately leads to the emergence of a large number of serious complications [6]. The pancreas or islet transplant remains the most reliable clinical approach to cure type 1 diabetes [7]. This problem prompted researchers to find an alternative solution that could have beneficial effects on the regulation of carbohydrate metabolism and to avoid the side effects of synthetic substances. Natural products used in alternative medicine are among the important axes of research of bioactive molecules that have a hypoglycemic effect [8].

Honey is a natural sweet substance formed by bees from the nectar of flowers or excretions left on plants by sucking insects (honeydew) [9]. There is an innumerable variety of honey, corresponding to the flowers and plants visited by the bees, as well as to the harvested source (nectar or honeydew). Thus, honey was separated into two categories: monofloral honey which comes predominantly from a single floral species (more than 45%) and polyfloral honey which results from bees harvesting from several floral species [10]. Generally, honey is composed of 60% to 85% of carbohydrates and 12% to 23% of water; it also contains enzymes, amino acids, organic acids, vitamins, and phenolic compounds [11].

Traditionally, honey was widely used for wound healing, as a food preservative, and against gastrointestinal illnesses [12]. According to recent literature, honey has revealed the presence of several beneficial effects for human health such as antibacterial [13], antioxidant, anti-inflammatory [14], and cardioprotective effects [15], with potentials as antiobesity [16] and neuroactivation associated with spatial memory [17].

Olive oil is an edible oil obtained from the cultivated varieties of *Olea europaea* belonging to the Oleaceae family [18]. It is a mixture of chemical compounds; it consists of more than 250 compounds whose saponifiable compounds (triglycerides, saturated, and unsaturated fatty acids) represent 98% or nonsaponifiable compounds 2% such as tocopherols (vitamin E), phenolic compounds, and pigments (chlorophylls, carotene) [19]. This composition changes according to the variety and to the pedoclimatic conditions [20].

The benefits of olive oil have been known for a long time; several studies have documented the pharmacological activities of this oil, for instance, antibacterial [21], anti-inflammatory [22], analgesic [23], and anticancer effects [24].

This study aims to evaluate the effect of olive oil and thyme honey as functional products widely used in complementary medicine, on diabetes type 1 induced by alloxan, and also to investigate the possible synergistic therapeutic effect between olive oil and thyme honey against diabetes type 1 and its complications such as dyslipidemia and hepatorenal dysfunction.

## 2. Materials and Methods

### 2.1. Thyme Honey and Olive Oil Samples

Monofloral thyme honey (*Thymus vulgaris.* L) and Moroccan Picholine variety of olive oil (*Olea europaea.* L) were purchased from Sefrou (latitude: 33.8305° N; longitude: 4.8353°W; altitude: 850 m; pluviometry: 3 to 14 mm; temperature: 7.7 to 25.6°C). Thyme honey was collected in July 2018 by a professional beekeeper, while olive oil was produced in January 2019 and stored in amber-colored bottles.

In this study, we evaluated the separate and combined effects of thyme honey and olive oil against type 1 diabetes induced by alloxan monohydrate. The choice of these natural products is based on their uses as complementary medicine [25–27].

### 2.2. Bioactive Compounds and Free Radical Scavenging Activity

#### 2.2.1. Polyphenols Content

Polyphenol quantification was determined as follows: 100 *μ*l of olive oil or 100 *μ*l of thyme honey solution (5 g of honey dissolved in 10 ml of distilled water) was mixed with 500 *μ*l of Folin-Ciocalteau (0.2 N) reagent and 400 *μ*l of sodium carbonate solution. Gallic acid was used as a standard to achieve the calibration curve; tests were made in triplicate, and the results were expressed as mean ± SD in mg gallic acid equivalent per 100 g sample (GA mg/100 g) [28, 29].

#### 2.2.2. Flavonoids Content

Flavonoid quantification was determined as follows: 100 *μ*l of olive oil or 100 *μ*l of thyme honey solution (5 g of honey dissolved in 10 ml of distilled water) were mixed with sodium nitrite (5%) and 150 *μ*l of AlCl_3_ solution 10%, 200 *μ*l of NaOH (1%) 1 M was added after 5 min, and the absorbance was measured at 510 nm. Tests were made in triplicate, and the results were expressed as mean ± SD. Flavonoid's content values were expressed as in milligrams of quercetin equivalent per 100 grams of the sample (QE mg/100 g) [28, 29].

#### 2.2.3. Content of Thyme Honey in Ascorbic Acid

The content of thyme honey in ascorbic acid was quantified following the titration method described by Nweze et al. Tests were made in triplicate, and the results were expressed as mean ± SD in mg/100 g [30].

#### 2.2.4. Free Radical Scavenging Activity

The method described by Miguel et al. was used for the determination of the DPPH radical scavenging activity [31]. Briefly, 100 *μ*l of olive oil or 25 *μ*l of thyme honey solution (5 g of honey dissolved in 10 ml of distilled water) were mixed with 875 *μ*l of DPPH solution (63.4 *μ*M). The absorbance was read at 517 nm, and the antiradical activity was estimated based on the percentage of DPPH radical scavenged using the following formula:

(1) $$\begin{array}{c} \text{IC}50\%\;=\left[\frac{\left(\text{control absorbance}-\text{sample absorbance}\right)}{\text{control absorbance}}\right]\times 100. \end{array}$$

Tests were made in triplicate, and the results were expressed as mean ± SD in mg/ml.

### 2.3. Phenolics Compounds Identification and Quantification in Olive Oil and Thyme Honey

The analysis of the polyphenols in the olive oil sample was carried out on a SHIMADZU PROMINENCE HPLC system fitted with a DAD detector, a degasser, and an LC A20 type pump as well as an RYODINE type manual injector. The separation by HPLC of the polyphenols was carried out on an Agilent Zorbax C18 column dimension 4.6 mm × 250 mm 5 um 100 A at A flow rate of 1 ml/min of a ternary mobile phase of acetonitrile, methanol, and water; column temperature was 30°C; and the injection volume was 20 *μ*l. Under the same conditions, syringic acid and Tyrosol standard solutions were injected to determine the response factor [32]. The phenolic compounds in thyme honey have been analyzed previously [33].

### 2.4. Mineral Content in Thyme Honey and Olive Oil

Mineral contents in thyme honey and olive oil were analyzed by the calcination method using ICP-AES, following the method described by Silva et al. Briefly, 5 ml of nitric acid 0.1 M were added to the ashes of honey and olive oil. Then, 10 ml of the same acid was added, and the mixture was made up to 25 ml with ultrapure water; mineral elements were determined using an air/acetylene flame, and the quantitative determination was carried out after calibrating the instrument using ranges of calibrations of Na, K, Ca, Mg, Fe, Cu, Zn, Ni, Cd, and Pb dissolved in 0.1% lanthanum. All samples were analyzed in triplicate [34].

### 2.5. Physicochemical Analysis of Thyme Honey

#### 2.5.1. Electrical Conductivity

Twenty grams of thyme honey was dissolved in 100 ml of distilled water, and then the electrical conductivity was measured at 20°C using an electrical conductivity cell [35].

#### 2.5.2. pH

Ten grams of thyme honey was dissolved in 100 ml of ultrapure water using a pH meter [35].

#### 2.5.3. Free Acidity and Lactone Acidity

Free acidity assay was determined as follows: 1 g of thyme honey was dissolved in 25 ml of ultrapure water and a solution of NaOH 0.05 M up to the equivalence point (pHe = 8.3). Lactone acidity was obtained by the addition of 1 ml of NaOH 0.05 M followed by titration with HCl 0.05 to return to the equivalence point [35].

#### 2.5.4. Ash Content

The ash content was obtained by ashing 5 g of thyme honey at 600°C, and then the weight of ash was measured [35].

#### 2.5.5. Moisture and Total Soluble Solids (TSSs)

The refractometer was used for moisture and total soluble solids analysis [29].

#### 2.5.6. Diastase Activity

Diastase activity was analyzed as described by Bogdanov and then calculated using formula (1): diastase number=300/*Tx*. Tx is the time taken by the reaction for the absorbance of the blue color to decrease to approximately 0.235 [35].

#### 2.5.7. Thyme Honey Color

Ten grams of honey was dissolved in 20 ml of distilled water; then the absorbance was measured at 635 nm using a spectrophotometer. The mm *P*fund values were obtained using the following formula [36]:

(2) $$\begin{array}{c} mm\;P\text{fund}=-38.7+371.39\times \text{absorbances.} \end{array}$$

#### 2.5.8. Melanoidins Content

Melanoidins content was estimated based on the browning index by measuring the net absorbance of thyme honey at 450 nm and 720 nm (net absorbance = A450 − A720) [37].

### 2.6. Physicochemical Analysis of Olive Oil

#### 2.6.1. Free Acidity

The free acidity, expressed as a percentage of oleic acid, was determined by dissolving 1 g of olive oil in a diethyl ether/ethylic alcohol solution 1 : 1. The mixture was titrated with a 0.1 N NaOH solution [38].

#### 2.6.2. Peroxide Index

One gram of olive oil was dissolved in 12.2 ml of the acid mixture acetic/chloroform 3 : 2 (v/v). The mixture obtained was titrated with a 0.01 N sodium thiosulfate solution [38].

#### 2.6.3. Specific Extinction Coefficient at 232 nm and 270 nm

The specific extinction coefficient at 232 nm and 270 nm was determined according to the protocol described by the official analytical methods in EC Regulation 2568/91 [39].

#### 2.6.4. Chlorophyll Content

Here, 7.5 g of olive oil was dissolved in cyclohexane and taken to a final volume of 25 ml. The absorbance of the mixture was determined at 470 nm, the extinction coefficient applied was *E*_0_ = 613, and chlorophyll content was calculated using the following formula:

(3) $$\begin{array}{c} \text{chlorophyll content}=\frac{\left(A\;670\times 106\right)}{\left(613\times 100\times d\right)}, \end{array}$$

*A* is the absorbance, and *d* is the spectrophotometer cell thickness (1 cm). The tests were made in triplicate, and the results were expressed as mean ± SD in mg/kg [40].

#### 2.6.5. Carotenoids Content in Olive Oil

Here, 7.5 g of olive oil was dissolved in cyclohexane and taken to a final volume of 25 ml. The absorbance of the mixture was determined at 470 nm, the extinction coefficient applied was *E*_0_ = 2000, and the carotenoids content was calculated using the following formula:

(4) $$\begin{array}{c} \text{carotenoids content}=\frac{\left(A470\times 106\right)}{\left(2000\times 100\times d\right)}, \end{array}$$

*A* is the absorbance, and *d* is the spectrophotometer cell thickness (1 cm). The tests were made in triplicate, and the results were expressed as mean ± SD in mg/kg [40].

### 2.7. Induction of Diabetes Mellitus and Experimental Design

#### 2.7.1. Ethical Approval

The ethical approval was obtained from Sidi Mohamed Ben Abdellah University in Fez under the responsibility of the Laboratory of Natural Substances, Pharmacology, Environment, Modeling, Health, and Quality of life (SNAMOPEQ), Department of Biology, Faculty of Sciences Dhar EL Mahraz of Fez, Morocco (L.20.USMBA-SNAMOPEQ 2017-03). The care and handling of the animals were following the internationally accepted standard guidelines for the Care and Use of Laboratory Animals, and the protocol was approved by our institutional committee on animal care, University Sidi Mohamed Ben Abdellah, Faculty of Sciences Dhar EL Mahraz Fez, Morocco.

#### 2.7.2. Diabetes Induction

A total of 42 rats with 190.85 ± 5.14 g of body weight (BW) were randomly divided into 7 groups, with 6 rats in each group. The animals were housed in a standard environmental condition (23 ± 3°C with 12 h light/dark cycles) and fed with standard rodent chow and water ad libitum. Diabetes mellitus was induced in overnight fasted healthy male Wistar rats by a single intravenous injection of alloxan monohydrate (65 mg/kg BW) prepared in normal saline. After 72 h of alloxan injection, only rats with glycemia ≥250 mg/dl were considered diabetics and used in this study.

The experimental protocol was carried out as follows:

  Group1 (DW): six healthy rats (nondiabetic) received distilled water (10 ml/kg BW)
  Group2 (TH): six healthy rats (nondiabetic) received thyme honey (2 g/kg BW)
  Group3 (OO): six healthy rats (nondiabetic) received olive oil (10 ml/kg BW)
  Group4 (DC + DW): six diabetic rats were treated with distilled water (10 ml/kg BW)
  Group5 (DC + TH): six diabetic rats were treated with thyme honey (2 g/kg BW)
  Group6 (DC + OO): six diabetic rats were treated with olive oil (10 ml/kg BW)
  Group7 (DC + TH + OO): six diabetic rats were treated with a combination of thyme honey (1 g/kg BW) and olive oil (5 ml/kg BW)

All treatments were given orally every day for 30 days; the rats were weighed every ten days. At the end of the experiment, the urine of each rat was collected and the blood was taken under diethyl ether anesthesia by retro-orbital bleeding, the samples were separated by centrifugation (2000 ×g) for 10 min.

#### 2.7.3. Biochemical Analysis

Serum samples were analyzed for blood fasting glucose, hepatic enzymes (aspartate aminotransferases (AST), alanine aminotransferases (ALT), alkaline phosphatase (ALP), lactate dehydrogenase (LDH)), lipidic profile (total cholesterol (TC), triglycerides (TG), high-density lipoprotein (HDL-C), low-density lipoprotein (LDL-C)), and kidney parameters (urea, uric acid, creatinine, total protein, sodium (Na^+^), potassium (K^+^), chloride (Cl^−^)).

Urine samples were analyzed for uric acid, creatinine, total protein, sodium (Na^+^), potassium (K^+^), and chloride (Cl^−^).

#### 2.7.4. Catalase and Glutathione Peroxidase Activity in the Pancreas, Liver, and Kidneys of Normal and Diabetic Rats

Catalase (CAT) activity was calculated according to the method described by Aebi et al. [41]. A decrease in absorbance due to H_2_O_2_ degradation was monitored spectrophotometrically at 240 nm for 1 min, and the activity was expressed as *μ*mol H_2_O_2_/min/mg protein. Glutathione peroxidase (GPx) activity was estimated according to the method of Flohé [42]. The activity was expressed as moles of GSH oxidized/min/mg protein.

#### 2.7.5. Reduced Glutathione (GSH) Levels in the Pancreas, Liver, and Kidneys of Normal and Diabetic Rats

GSH levels were measured following the protocol described by Ellman [43]. Briefly, 3 mL of sulfosalicylic acid (4%) was added to 500 mL of the homogenate liver, pancreas, and kidney tissues. The mixture was centrifuged at 2,500 ×g for 15 min, and then prepared Ellman's reagent was added to 500 mL of supernatant. The absorbance was measured at 412 nm after 10 min. The total GSH content was expressed as *μ*g/g of tissue.

#### 2.7.6. Lipid Peroxidation (MDA) Levels in the Pancreas, Liver, and Kidneys of Normal and Diabetic Rats

The formation of products of lipid peroxidation was quantified in the liver, pancreas, and kidney tissues using the thiobarbituric acid–reactive substances (TBARSs) method, as reported previously by Kassan [44], and absorbance was measured at 532 nm. Results were expressed as malondialdehyde (MDA) concentration (nmol/g tissue).

#### 2.7.7. Histological Analysis

Histological analysis of the liver, pancreas, and kidney was performed using the method described by Bakour et al. [28]. The organs were fixed in the formalin solution (10%) for 24 h, and then tissue samples were dehydrated using ethanol with a series of increasing concentrations. The organs were next clarified in toluene and then embedded in paraffin. A microtome was used to cut fine sections (5–6 mm) from paraffin blocks. Hematoxylin and Eosin (H&E) were used for staining the slides obtained for observation under an optical microscope.

### 2.8. Statistical Analysis

Statistical comparisons between the groups were performed with one-way analysis of variance (ANOVA) followed by the Tukey test, and the comparison between rat BWs was made by *t*-test using GraphPad Prism ® software (version 5.0; GraphPad Software, Inc., San Diego, USA). Data were represented as mean ± SD (^*∗*^*p* < 0.05, ^∗∗^*p* < 0.01, and ^∗∗∗^*p* < 0.001).

## 3. Results and Discussion

### 3.1. Bioactive Compounds and Free Radical Scavenging Activity

Antioxidants are bioactive molecules present in food in varying concentrations; they protect the body's cells against the harmful effects of free radicals, and they prevent the organism from several diseases [45]. There is compelling evidence that consuming foods rich in antioxidants like polyphenols, flavonoids, and vitamins is strongly associated with good health and functional longevity [46]. In the present study, the antioxidant content in thyme honey and olive oil was analyzed, and the results revealed that thyme honey contains a high concentration of antioxidant compounds (polyphenols: 192.82 ± 15.24 GA·mg/100 g; flavonoids: 11.59 ± 0.20 QE·mg/100 g and ascorbic acid: 13.16 ± 1.22 mg/100 g), as well as a good free radical scavenging activity (IC_50_ = 15.37 ± 0.97 mg/ml) (Table 1). These results are in the range of those found by Laaroussi et al. for eight samples of *Bupleurum spinosum* honey [29] and almost similar to the findings obtained by Bouhlali et *al* for Moroccan thyme honey with a polyphenol content of 113.853 ± 1.103 GA·mg/100 g and flavonoids content of 17.908 ± 0.137 rutin equivalents mg/100 g [47]. The antioxidant activity of the studied thyme honey was evaluated using the DPPH free radical scavenging activity, and the concentration of thyme honey required to scavenge 50% of DPPH free radicals was (IC_50_ = 15.37 ± 0.97 mg/ml). The antioxidant activity of honey is highly related to the floral source and the pedoclimatic conditions [48].

*In vitro* tests of olive oil showed a content of 33.48 ± 1.54 GA·mg/100 g in polyphenols and 16.03 ± 0.56 QE mg/100 g in flavonoids. The results of the DPPH test showed an IC_50_ value of 2.74 ± 0.12 mg/mL (Table 1). These results were better than those obtained by Negro et al. for eight genotypes/cultivars from Italy (Colozzese, Barone di Monteprofico, Cellina di Nardò, Cornola, Ogliarola di Lecce, Orniella, Oliva Grossa, Spina) [49]. Several factors can influence the content of phenolic compounds in olive oil, such as olive maturation, seasonal variation, environmental factors, the intravarietal diversity of the olive tree, and the method of extraction. The rich composition of our sample studied in polyphenols revealed that the olive oil studied did not undergo any heating and it was stored and handled in very good condition. These compounds, like the other minor constituents of olive oil, contribute to sensory and organoleptic properties and the prevention of oil auto-oxidation [50].

### 3.2. Antioxidant Compounds Identification and Quantification in Olive Oil

Different classes of antioxidants are present in olive oil such as phenolic alcohols, phenolic acids, secoiridoids and derivatives, hydroxycinnamic acid derivatives, flavonoids, triterpenic acids, and tocopherols [51]. The sensory properties and health benefits of olive oil are strongly linked to its volatile and phenolic composition [52]. Certain compounds, such as oleuropein, give olive oil a bitter flavor and a pungent sensation [53]. The analysis of antioxidant compounds found in our studied olive oil is presented in Table 2 and revealed that the predominant compound is oleacin (253.80 ± 1.52 mg/kg), followed by tyrosol (67.04 ± 1.12 mg/kg), hydroxytyrosol (36 ± 1 mg/kg), apigenin (31 ± 1 mg/kg), and syringic acid (15 ± 1 mg/kg). The lowest concentrations showed by luteolin, oleuropein, and cinnamic acid are 7.20 ± 0.14 mg/kg, 4.86 ± 0.63 mg/kg, and 3.35 ± 0.11 mg/kg, respectively. The secoiridoids present in olive oil are derived mainly from oleuropein by enzymatic hydrolysis carried out by the *ß* glucosidase enzyme [54].

### 3.3. Mineral Content in Thyme Honey and Olive Oil

Macroelements and microelements play an essential role in the metabolism and physiological functions of the organism. Adequate levels of mineral elements are essential to cope with the metabolic responses linked to the pathological situation. Micronutrients such as minerals are provided mainly by foods [55]. The results of mineral analysis of thyme honey and olive oil studied showed that the content in macroelements in both samples was in the following order: the major element was sodium (954.24 ± 0.20 mg/kg in olive oil and 1012.32 ± 0.12 mg/kg in thyme honey) followed by potassium (405.94 ± 0.30 mg/kg in olive oil and 752.63 ± 0.52 mg/kg in thyme honey), calcium (204.05 ± 1.12 mg/kg in olive oil and 124.28 ± 0.18 mg/kg in thyme honey), magnesium (172.90 ± 1.63 mg/kg in olive oil and 85.31 ± 0.59 in thyme honey), and iron (13.269 ± 0.86 mg/kg in olive oil and 1.58 ± 0.52 mg/kg in thyme honey). Similarly, for microelements, the major metals were zinc in thyme honey and olive oil with concentrations 7.98 ± 0.45 mg/kg and 5.0614 ± 0.52 mg/kg, respectively, followed by copper and nickel (Table 3). However, heavy metals like cadmium and lead are present in acceptable concentrations [56].

### 3.4. Physicochemical Parameters of Thyme Honey and Olive Oil

#### 3.4.1. Physicochemical Parameters of Thyme Honey

Physicochemical analyses of thyme honey are presented in Table 4. The honey analyzed in our study is acidic with a pH value of 4, which is in the range of Codex Alimentarius Commission standards [57]; the acidity of honey is due to its organic acid content [58].

Acidity is an important criterion of the quality of honey; the studied sample revealed a free acidity of 28.56 ± 1.54 meq/kg and a lactonic acidity of 9.13 ± 0.18 mEq/kg, with the total acidity value of 37.69 ± 1.46 meq/kg. These results are in line with those indicated by the Codex Alimentarius [57]. This indicates an absence of fermentation and the freshness of the studied honey.

The determination of water content (moisture) in honey allowed us to know the handling conditions of honey by beekeepers, such as storage, fermentation of honey, climate, and extraction conditions [29]. The analysis result shows that the water content of our sample is 19.0 ± 0.04% which remains below the maximum threshold recommended (20%) by the Codex Alimentarius [57].

The determination of the electrical conductivity and the ash content in honey allowed us to know the botanical origin of honey (monofloral or multifloral) and the mineral content of nectar [35]. Examination of the results of our sample shows that the electrical conductivity has a value of 462 *μ*S/cm, which is below the limit set by the Codex Alimentarius and EU Council (800 *μ*S/cm) [57, 59].

The color allows the classification of different kinds of honey according to the Pfund index. Our sample has a Pfund index of 121.74 ± 5.43 mm, and it is classified as a dark “Amber” color. Melanoidins are polymeric structures formed when sugar and amino acids combine as a result of the Maillard reaction; melanoidins analysis in thyme honey shows content of 0.83 ± 0.07. These results are in line with the recommended values cited by the United States Standards for Grades of Extracted Honey (USDA) [60].

The diastatic activity is an index of freshness and heat treatment of honey. For our sample, we found a value of 15.43 ± 2.03 (shading units/g) which complies with the standard set at a value greater than 8 by the EU Council [59]. This confirms that our honey has not been overheated, is fresh, and has been stored very well.

The TSS percentage in thyme honey was 81.0 ± 0.21. According to the United States Standards for Grades of Extracted Honey (USDA) [60], if the value of TSS was more than 80%, we can classify the honey sample as high grade and can be highly stable during storage.

#### 3.4.2. Physicochemical Parameters of Olive Oil

Physicochemical analyses of olive oil are presented in Table 4. Free acidity is a quality factor of olive oil, the result of free acidity in our analyzed sample was 0.56 ± 0.01%. According to the criteria cited by the Commercial Standard of the International Olive Oil Council, the olive oil analyzed is classed as the extra virgin type (acidity less than 0.8) [61].

The peroxide index in olive oil was 4.6 ± 0.04 mEq O_2_/kg; this value is within the limits established by the commercial standard of the International Olive Oil Council (≤20mEq O_2_/kg) [61].

The values of specific ultraviolet extinctions K232 and K270 were 0.08 ± 0.00 and 0.21 ± 0.00, respectively. These values do not exceed the limits established by the commercial standard of the International Olive Oil Council (K232 = 2.60 and K270 = 0.25) [61].

The chlorophyll and carotenoids contents were 14.55 ± 1.02 mg/kg and 6.2 ± 0.7 mg/kg, respectively.

### 3.5. Effect of Thyme Honey, Olive Oil, and Their Combination on Blood Glucose Level

In the present study, the protective effect of thyme honey and olive oil was investigated against type 1 diabetes; it is a genetically determined disease that causes low or no insulin production in the body, and subsequently, an increase in the blood glucose level [25].  Table 5 shows that after a single intravenous injection of alloxan monohydrate (65 mg/kg BW), the blood glucose level was increased significantly in rats injected compared to the groups of rats not injected by alloxan. The treatment of diabetic rats with thyme honey significantly decreased the blood glucose from (320 ± 20.19 mg/dl) on day 0 to (240.14 ± 30.89 mg/dl) on day 30; similarly, the treatment with olive oil decreased the glycemia from 359 ± 19.79 mg/dl on day 0 to 163.5 ± 21.92 mg/dl in day 30. Interestingly, the combination of thyme honey and olive oil significantly decreased the blood glucose level from (318 ± 30.21 mg/dl) on day 0 to (158.54 ± 20.42 mg/dl) on day 30. The hypoglycemic effect of thyme honey may be related to its phenolics composition (gallic acid (2.86 ± 0.046 mg/100 g); ferulic acid (0.68 ± 0.021 mg/100 g), caffeic acid (0.033 ± 0.00 mg/100 g), epicatechin gallate (6.91 ± 0.05 mg/100 g), and pyrogallol (3.5 ± 0.009 mg/100 g)) [33]. Huang et al. have shown that gallic acid can ameliorate hyperglycemia and improves hepatic carbohydrate metabolism in diabetes induced by high-fructose diet in rats [62]. Similarly, it has been shown that caffeic acid has an antidiabetic effect via the inhibition of *α*-amylase and *α*-glucosidase [63] as well as epicatechin gallate supplementation can alleviate diabetes and lowered the glycemia level [64]. In addition, it is suggested that the fructose content of honey may contribute to the hypoglycemic effect of honey [65].

Many studies related the hypoglycemic effect of olive oil with its phenolic constituents. It has been proved that oleuropein, hydroxytyrosol, tyrosol, syringic acid, cinnamic acid, luteolin, and apigenin found in our olive oil sample (Table 2) have an important hypoglycemic effect [66–70].

### 3.6. Effect of Thyme Honey, Olive Oil, and Their Combination on Body Weight of Diabetic and Nondiabetic Rats

In the absence of insulin, the glucose is accumulated in the blood because it is neither stored in the liver nor used by the body's cells. At this condition, the body cells try to find another alternative to glucose using proteins and fats to produce the energy necessary for its functioning, this explains the weight loss in type 1 diabetics [70]. The change in body weight in diabetic rats and nondiabetic rats is shown in Table 6; the results revealed that the body weight of diabetic rats is significantly decreased, while in rats treated with thyme honey, olive oil, or by their combination, the body weight reduction statistically was not significant.

### 3.7. Effect of Thyme Honey, Olive Oil, and Their Combination on Hepatic Enzymes Level of Diabetic and Nondiabetic Rats

**Figure 1:**
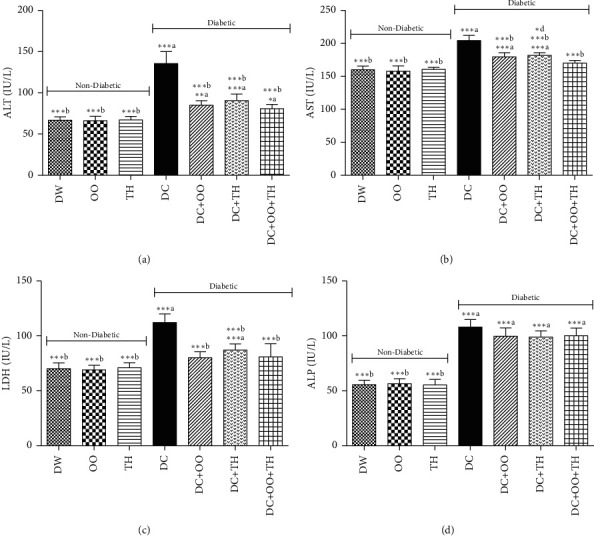
Effect of daily administration of thyme honey, olive oil, and their combination on hepatic enzymes level of diabetic and nondiabetic rats. Data are presented as mean ± SD; (a) comparison between the distilled water group (DW) and all groups; (b) comparison between the diabetic nontreated group (DC) and all groups; (c) comparison between the diabetic group treated by olive oil (DC + OO) and the diabetic group treated by thyme honey (DC + TH); (d) comparison between the diabetic group treated by the combination between olive oil and thyme honey (DC + OO + TH) and the groups treated by olive oil only (DC + OO) or thyme honey only (DC + TH).

The effect of thyme honey, olive oil, and their combination on the hepatic enzyme level of diabetic and nondiabetic rats is presented in Figure 1; the results showed that all hepatic enzymes (ALP, AST, LDH, ALP) were significantly increased in diabetic non-treated rats, while in the groups of rats treated with olive oil, thyme honey, or their combination, the rats were protected against the increase of AST, ALP, and LDH, and they are not protected against the elevation of ALP enzymes. The increase of the hepatic enzymes is due to the liver damage induced by diabetes toxicity and the leakage of these enzymes into the bloodstream [71]. The protective effect of olive oil and thyme honey is probably due to their phenolics content [72].

### 3.8. Effect of Thyme Honey, Olive Oil, and Their Combination on Lipid Profile of Diabetic and Nondiabetic Rats

**Figure 2:**
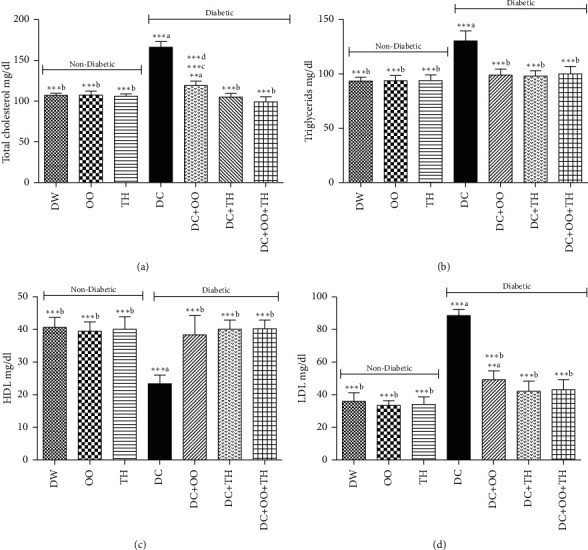
Effect of daily administration of thyme honey, olive oil, and their combination on the lipidic profile of diabetic and nondiabetic rats. Data are presented as mean ± SD; (a) comparison between the distilled water group (DW) and all groups; (b) comparison between the diabetic nontreated group (DC) and all groups; (c) comparison between the diabetic group treated by olive oil (DC + OO) and the diabetic group treated by thyme honey (DC + TH); (d) comparison between the diabetic group treated by the combination between olive oil and thyme honey (DC + OO + TH) and the groups treated by olive oil only (DC + OO) or thyme honey only (DC + TH).

Dyslipidemia is among the most well-known complications of diabetes; it is highly related to the risk of cardiovascular disease [73].  Figure 2 presents the effect of the daily administration of thyme honey, olive oil, and their combination on the lipid profile in diabetic and nondiabetic rats. The results showed that the dose of alloxan injection caused type 1 diabetes and led to dyslipidemia (the increase in total cholesterol, triglycerides, LDL, and decrease in HDL levels). The same results were obtained by others authors using alloxan or streptozotocin as diabetogenic agents [74, 75]. The groups of rats treated with thyme honey, olive oil, or their combination have protected the diabetic rats against this metabolic disorder in a significant manner (*p* < 0.001).

### 3.9. Effect of Thyme Honey, Olive Oil, and Their Combination on Kidney Parameters of Diabetic and Nondiabetic Rats

**Figure 3:**
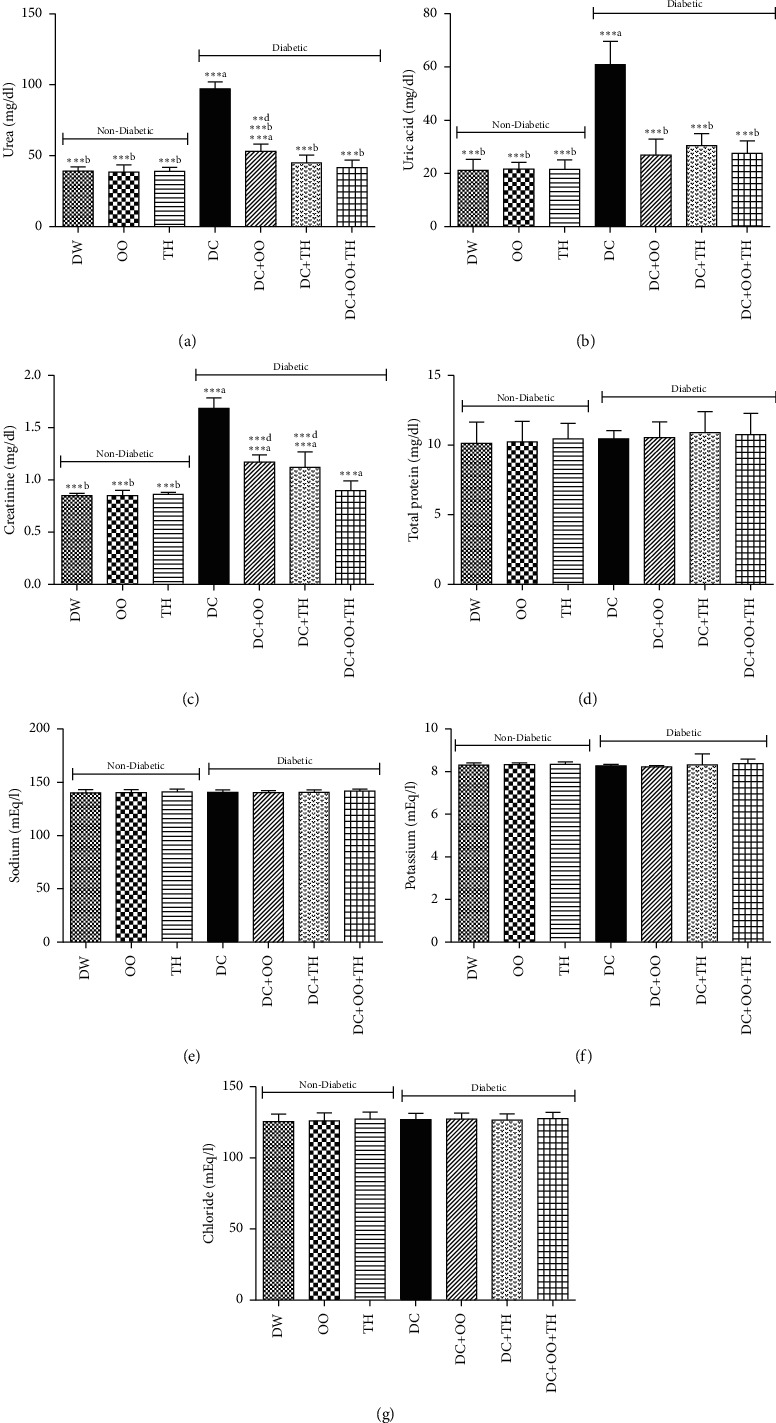
Effect of daily administration of thyme honey, olive oil, and their combination on serum kidney parameters of diabetic and nondiabetic rats. Data are presented as mean ± SD; (a) comparison between the distilled water group (DW) and all groups; (b) comparison between the diabetic nontreated group (DC) and all groups; (c) comparison between the diabetic group treated by olive oil (DC + OO) and the diabetic group treated by thyme honey (DC + TH); (d) comparison between the diabetic group treated by the combination between olive oil and thyme honey (DC + OO + TH) and the groups treated by olive oil only (DC + OO) or thyme honey only (DC + TH).

**Figure 4:**
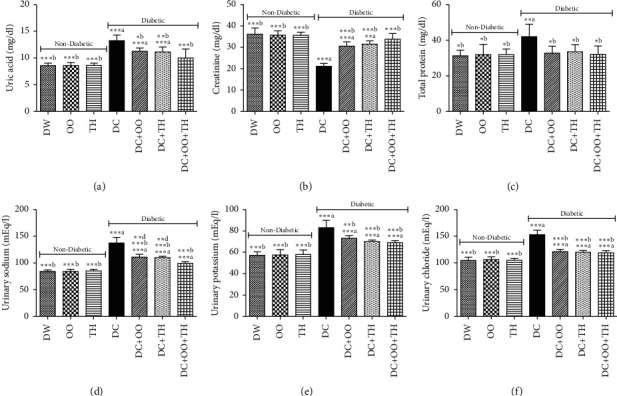
Effect of daily administration of thyme honey, olive oil, and their combination on urinary kidney parameters of diabetic and nondiabetic rats. Data are presented as mean ± SD; (a) comparison between the distilled water group (DW) and all groups; (b) comparison between the diabetic nontreated group (DC) and all groups; (c) comparison between the diabetic group treated by olive oil (DC + OO) and the diabetic group treated by thyme honey (DC + TH); (d) comparison between the diabetic group treated by the combination between olive oil and thyme honey (DC + OO + TH) and the groups treated by olive oil only (DC + OO) or thyme honey only (DC + TH).

Kidney dysfunction is known as a complication of diabetes [76]. Figures 3 and 4 summarize the results of the effect of thyme honey, olive oil, and their combination on kidney parameters analyzed in urine and serum of diabetic and nondiabetic rats. The results revealed an increase in the serum levels of urea, uric acid, and creatinine in diabetic rats nontreated compared to the values obtained in control rats, while no change was recorded in total protein, potassium, sodium, and chloride levels. The treatment of diabetic rats with olive oil, thyme honey, or their combination has a protective effect against the elevation of urea, uric acid, and creatinine in diabetic treated rats. For the urine analysis, the injection of alloxan leads to an increase in urine uric acid, creatinine, total protein, and the urinary excretion of sodium, potassium, and chloride. It was revealed that the main cause of diabetic kidney toxicity is oxidative stress. Thereby, supplementation with phenolic acids may be effective against this toxicity [77].

### 3.10. Effect of Thyme Honey, Olive Oil, and Their Combination on the Kidney, Pancreas, and Liver Enzymatic Antioxidants and Lipid Peroxidation of Diabetic and Nondiabetic Rats

Oxidative stress is known as an imbalance between free radicals and antioxidants in the body [78]. Several studies proved that diabetes is accompanied by oxidative stress and increased free radical formation [79, 80]. Tables 7–9 summarize the effect of thyme honey, olive oil, and their combination on the kidney, pancreas, and liver enzymatic antioxidants of diabetic and nondiabetic rats. The results presented in these Tables showed that in the three organs (kidney, liver, and pancreas), the levels of proteins, catalase, GSH, and GPx were significantly decreased (*p* < 0.001), while the MDA was increased in the same manner in diabetic nontreated rats. In the treated rats whether, by thyme honey, olive oil, or by their combination, we observed a significant increase in enzymatic antioxidant levels and a decrease in MDA levels. Increasing enzymatic and nonenzymatic antioxidants in the body is the most important strategy to prevent lipid peroxidation and to fight oxidative stress which is the main cause of the onset of diabetic complications [81].

### 3.11. Effect of Thyme Honey, Olive Oil, and Their Combination on the Liver, Kidney, and Pancreas Tissues of Diabetic and Nondiabetic Rats

**Figure 5:**
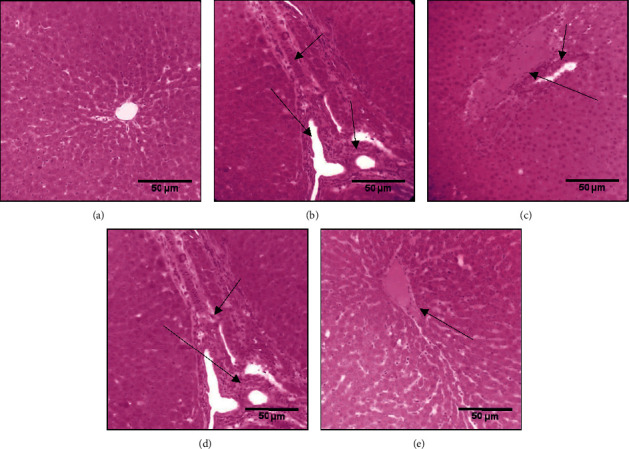
Effect of thyme honey, olive oil, and thyme combination on the liver tissue of diabetic and nondiabetic rats. (a) DW group, (b) diabetic untreated group (DC), (c) diabetic group treated with olive oil, (d) diabetic group treated with thyme honey, and (e) diabetic group treated with thyme honey and olive oil.

Liver histological analysis is illustrated in Figure 5. The liver of the nondiabetic group that received distilled water only revealed normal microscopic architecture formed by Pacini hexagonal lobules which centered on the central vein (Figure 5(a)). In the diabetic untreated group (Figure 5(b)), the liver structure revealed congestion of the central vein with disorganization of hepatic architecture, which could induce severe destruction and necrosis. The administration of honey and olive oil at the dose of 2 mg/kg and 10 mg/kg, respectively, or combined exhibited a morphology near to that of the control structure for olive oil (Figure 5(c)). The combination of olive oil and honey ameliorate congestion of the hepatic artery, mild dilatation of portal vein, and moderate disorganization of hepatic architecture (Figure 5(d)), while thyme honey treatment revealed a modest ability to counteract the alloxan's hepatic injuries (Figure 5(c)).

**Figure 6:**
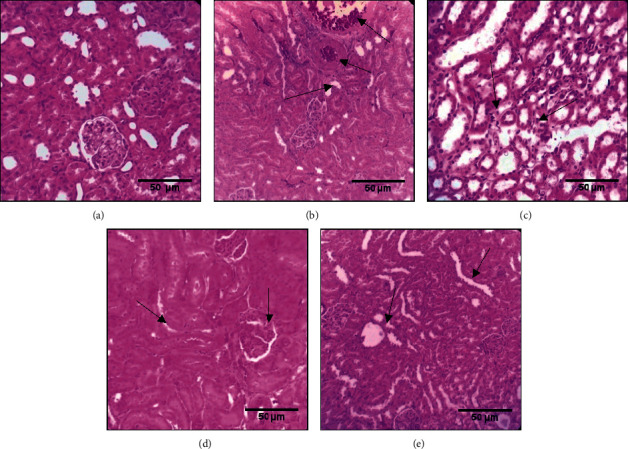
Effect of thyme honey, olive oil, and thyme combination on kidney tissue of diabetic and nondiabetic rats. (a) DW group, (b) diabetic untreated group (DC), (c) diabetic group treated with olive oil, (d) diabetic group treated with thyme honey, and (e) diabetic group treated with thyme honey and olive oil.

The histological analysis of the kidney was presented in Figure 6. The kidneys of diabetic untreated rats' revealed changes in the architecture (Figure 6(b)) as compared to the control group (Figure 6(a)). Furthermore, there is a decrease in Bowman's capsule space with disorganization of the kidney structure and renal vein dilatation. The renal tissue in groups treated with thyme honey or thyme honey/olive oil was well protected against injuries induced by alloxan (Figures 6(d) and 6(e)). While in the group treated with olive oil alone, the kidney histological examination showed a deposit of immune cells and vacuolar modification seen in the tubular cells (Figure 6(c)).

**Figure 7:**
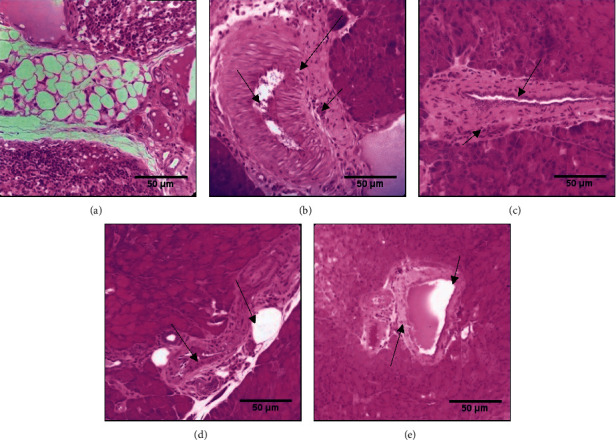
The effect of thyme honey, olive oil, and thyme combination on the pancreas tissue of diabetic and nondiabetic rats. (a) DW group, (b) diabetic untreated group (DC), (c) diabetic group treated with olive oil, (d) diabetic group treated with thyme honey, and (e) diabetic group treated with thyme honey and olive oil.

The histological analysis of pancreatic tissue is presented in Figure 7. The tissue of the untreated diabetic rats revealed necrotic changes induced by alloxan. It induced eosinophilia, increased cellular infiltration, hemorrhage, and disorganization of pancreatic structure (Figure 7(b)). However, the use of honey and olive oil alone or combined (Figures 7(c)–7(e)) protect relatively the pancreatic architecture by recovering a structure close to that showed in the normal group (Figure 7(a)).

The histopathological changes of the liver, kidney, and pancreas showed in the diabetic group were previously observed in other studies [82, 83] because these organs are the main targets of the complications induced by diabetes. Olive oil and thyme honey exhibit a protective effect against these changes induced by a single injection of alloxan. The same results were observed in other studies like those of Balamash et al. showing that virgin olive oil from Saudi Arabia has a protective effect against the histopathological changes of the liver, pancreas, and kidneys induced by streptozotocin in Sprague–Dawley rats [84]. Similarly, Afroz et al. have shown that honey has a protective effect on histological changes of the liver and kidneys induced by acetaminophen [85].

## 4. Conclusion

Overall, we can conclude from the results obtained in this study that thyme honey or olive oil, and especially, their combination improved significantly the blood glucose levels and protected against metabolic changes and the complications induced by type 1 diabetes. More in-depth studies are needed to know the molecules responsible for the pharmacological effect observed in this study, as well as to understand the exact mechanism of action.

## Figures and Tables

**Table 1:** Bioactive compounds and free radical scavenging activity of thyme honey and olive oil.

	Polyphenols GA·mg/100 g	Flavonoids QE·mg/100 g	Ascorbic acid (mg/100 g)	DPPH (IC_50_ = mg/mL)
Thyme honey	192.82 ± 15.24	11.59 ± 0.20	13.16 ± 1.22	15.37 ± 0.97
Olive oil	33.48 ± 1.54	16.03 ± 0.56	—	2.74 ± 0.12

**Table 2:** Phenolic compounds identification and quantification in olive oil.

Phenolic compounds	Concentration (mg/kg)
Hydroxytyrosol	36 ± 1
Tyrosol	67.04 ± 1.12
Syringic acid	15 ± 1
Oleuropein	4.89 ± 0.63
Oleacein	253.80 ± 1.52
Pinoresinol	24.04 ± 0.21
Cinnamic acid	3.351 ± 0.11
Luteolin	7.20 ± 0.14
Apigenin	31 ± 1
Total	442.321 ± 6.73

**Table 3:** Minerals content in thyme honey and olive oil.

Sample	Minerals (mg/kg)				
Olive oil	Na	K	Ca	Mg	Fe
	954.24 ± 0.20	405.94 ± 0.30	204.05 ± 1.12	172.90 ± 1.63	13.269 ± 0.86
	Cu	Zn	Ni	Cd	Pb
	0.438 ± 0.09	5.0614 ± 0.52	0.2357 ± 0.15	0.0903 ± 0.011	ND

Thyme honey	Na	K	Ca	Mg	Fe
	1012.32 ± 0.12	752.63 ± 0.52	124.28 ± 0.18	85.31 ± 0.59	1.58 ± 0.52
	Cu	Zn	Ni	Cd	Pb
	0.827 ± 0.124	7.98 ± 0.45	0.087 ± 0.012	0.047 ± 0.004	0.017 ± 0.01

**Table 4:** Physicochemical analysis of thyme honey and olive oil.

	Physicochemical analysis					
Thyme honey	pH	Free acidity (mEq/kg)	Lactonic acidity (mEq/kg)	Total acidity (mEq/kg)	Moisture (%)	Ash content (%)
	4.0 ± 0.05	28.56 ± 1.54	9.13 ± 0.18	37.69 ± 1.46	19.0 ± 0.04	0.27 ± 0.00
	Electrical conductivity (mS/cm)	Diastasic activity (shading units/g)	TSS (%)	Pfund scale (mm)	Honey color	Melanoidins
	462 ± 5.12	15.43 ± 2.03	81.0 ± 0.21	121.74 ± 5.43	Amber	0.83 ± 0.07

Olive oil	Free acidity %	Peroxide index mEq O_2_/kg	K232	K270	Chlorophyll (mg/kg)	Carotenoids (mg/kg)
	0.56 ± 0.01	4.6 ± 0.04	0.08 ± 0.00	0.21 ± 0.00	14.55 ± 1.02	6.2 ± 0.7

**Table 5:** Effect of daily oral administration of thyme honey, olive oil, and their combination on blood glucose level in the diabetic and nondiabetic rats.

Experimental groups	Blood fasting glucose levels (mg/dl)			
	Day 0 (baseline)	Day 10	Day 20	Day 30
DW	101 ± 4.24	97 ± 2.82	98.5 ± 4.94	102.5 ± 4.94^**≠≠≠c**^
OO	95.5 ± 6.36	96 ± 1.41	98 ± 5.65	97 ± 7.07^**≠≠≠c**^
TH	98 ± 2.82	99 ± 1.56	100 ± 1.12	98 ± 4.59^**≠≠≠c**^
DC	351.5 ± 27.57	399.5 ± 16.26^∗∗**a**^	417,5 ± 13.43^∗∗∗**a**^	437 ± 26.87^∗∗∗**a**≠≠≠**b**^
DC + OO	359 ± 19.79	263 ± 21.21^∗∗∗**a**^	201 ± 15.55^∗∗∗**a**^	163.5 ± 21.92^∗∗∗**a**≠≠≠**b**≠≠≠**c**^
DC + TH	320 ± 20.19	300 ± 60.12	261 ± 30.15	240.14 ± 30.89^∗∗**a**≠≠≠**b**≠≠≠**c**^
DC + OO + TH	318 ± 30.21	260.18 ± 42.40^*∗ ***a**^	200.80 ± 12.56^∗∗∗**a**^	158.54 ± 20.42^∗∗∗**a**≠≠≠**b**≠≠≠**c**^

Data are expressed as mean ± SD; a: comparison between day 0 and the other days, b: comparison between the control group (DW) and all groups on day 30, and c: comparison between the diabetic group (DC) and all groups on day 30 (^*∗*^*p* < 0.05, ^∗∗^*p* < 0.01, and ^∗∗∗^*p* < 0.001).

**Table 6:** Effect of daily oral administration of thyme honey, olive oil, and their combination on body weight.

Experimental groups	Body weight (g)		
	Day 0 (baseline)	Day 30	Body weight change
DW	180 ± 6	220 ± 10	+40 ± 4
OO	195 ± 3	221 ± 14^∗∗^	+26 ± 11
TH	190 ± 4	212 ± 9	+22 ± 5
DC	193 ± 5	125 ± 15^*∗*^	−68 ± 10
DC + OO	178 ± 7	165 ± 9	−13 ± 2
DC + TH	199 ± 7	170 ± 8	−29 ± 1
DC + OO + TH	201 ± 4	178 ± 7	−23 ± 3

Data are expressed as mean ± SD; the comparison between day 0 and day 30 was made using a *t*-test.

**Table 7:** Effect of daily administration of thyme honey, olive oil, and their combination on kidney enzymatic antioxidants of diabetic and nondiabetic rats.

Variables in the kidney	Interventions						
	DW	OO	TH	DC	DC + OO	DC + TH	DC + OO + TH
Proteins (mg/g org)	5.02 ± 0.5^∗∗∗**b**^	4.99 ± 0.66^∗∗∗**b**^	5.01 ± 0.46^∗∗∗**b**^	2.47 ± 0.53^∗∗∗**a**^	4.33 ± 0.57^∗∗∗**b**^	3.99 ± 0.18^*∗ ***a**∗∗∗**b**^	4.46 ± 0.19^∗∗∗**b**^
Catalase (*μ*mol H_2_O_2_/min/mg pr)	29.23 ± 1.41	29.21 ± 1.39^∗∗∗**b**^	29.22 ± 1.40^∗∗∗**b**^	8.90 ± 0.89^∗∗∗**a**^	27.60 ± 1.21^∗∗∗**b**^	26.36 ± 3.32^∗∗∗**b**^	28.79 ± 1.83^∗∗∗**b**^
GSH (*μ*g/g org)	209.09 ± 4.16^∗∗∗**b**^	208.12 ± 4.10^∗∗∗**b**^	209.11 ± 4.09^∗∗∗**b**^	60.50 ± 3.52^∗∗∗**a**^	188.65 ± 4.76^∗∗∗**a**∗∗∗**b**∗∗∗**c**∗∗∗**d**^	200.48 ± 1.01^∗∗∗**a**∗∗∗**b**^	206.51 ± 3.55^∗∗∗**b**^
GPx (nmol GSH/min/mg pr)	11.20 ± 0.66^∗∗∗**b**^	11.19 ± 0.67^∗∗∗**b**^	11.21 ± 0.59^∗∗∗**b**^	2.94 ± 0.31^∗∗∗**a**^	10.06 ± 0.81^∗∗∗**a**∗∗∗**b**^	10.13 ± 0.34^∗∗∗**a**∗∗∗**b**^	10.48 ± 0.58^∗∗∗**b**^
MDA (nmol/g org)	25.96 ± 1.01^∗∗∗**b**^	25.98 ± 0.98^∗∗∗**b**^	25.96 ± 0.99^∗∗∗**b**^	44.64 ± 1.50^∗∗∗**a**^	30.28 ± 2.00^∗∗∗**a**∗∗∗**b**^	28.09 ± 0.50^∗∗∗**b**^	28.42 ± 1.01^*∗ ***a**∗∗∗**b**^

Data presented as mean ± SD; a: comparison between distilled water group (DW) and all groups; b: comparison between diabetic nontreated group (DC) and all groups; c: comparison between the diabetic group treated by olive oil (DC + OO) and the diabetic group treated by thyme honey (DC + TH); d: comparison between the diabetic group treated by the combination between olive oil and thyme honey (DC + OO + TH) and the groups treated by olive oil only (DC + OO) or thyme honey only (DC + TH).

**Table 8:** Effect of daily administration of thyme honey, olive oil, and their combination on liver enzymatic antioxidants of diabetic and nondiabetic rats.

Variables in the liver	Interventions						
	DW	OO	TH	DC	DC + OO	DC + TH	DC + OO + TH
Proteins (mg/g org)	7.71 ± 0.57^∗∗∗**b**^	7.70 ± 0.56^∗∗∗**b**^	7.72 ± 0.56^∗∗∗**b**^	5.31 ± 0.28^∗∗∗**a**^	5.33 ± 0.19^∗∗∗**b**∗∗∗**c**∗∗∗**d**^	7.16 ± 0.11^∗∗∗**b**^	6.99 ± 0.18^∗∗∗**b**^
Catalase (*μ*mol H_2_O_2_/min/mg pr)	31.84 ± 0.8^∗∗∗**b**^	31.83 ± 1.03^∗∗∗**b**^	31.85 ± 0.91^∗∗∗**b**^	11.04 ± 1.67^∗∗∗**a**^	29.66 ± 0.31^∗∗∗**b**^	30.00 ± 1.60^∗∗∗**b**^	30.98 ± 2.15^∗∗∗**b**^
GSH (*μ*g/g org)	412.44 ± 4.94^∗∗∗**b**^	412.33 ± 4.99^∗∗∗**b**^	411.98 ± 5.18^∗∗∗**b**^	236.47 ± 6.01^∗∗∗**a**^	408.44 ± 5.33^∗∗∗**b**^	410.44 ± 5.28^∗∗∗**b**^	409.73 ± 6.94^∗∗∗**b**^
GPx (nmol GSH/min/mg pr)	14.47 ± 0.31^∗∗∗**b**^	14.48 ± 0.28^∗∗∗**b**^	14.50 ± 0.21^∗∗∗**b**^	9.93 ± 0.63^∗∗∗**a**^	10.83 ± 0.87^∗∗∗**a**^	9.88 ± 0.54^∗∗∗**a**∗∗∗**d**^	11.69 ± 1.11^∗∗∗**a**∗∗∗**b**^
MDA (nmol/g org)	38.81 ± 0.55^∗∗∗**b**^	38.18 ± 0.60^∗∗∗**b**^	37.28 ± 0.80^∗∗∗**b**^	77.88 ± 2.03^∗∗∗**a**^	41.40 ± 1.52^*∗ ***a**∗∗∗**b**^	39.75 ± 0.50^∗∗∗**b**^	40.63 ± 1.52^∗∗∗**b**^

Data presented as mean ± SD; a: comparison between distilled water group (DW) and all groups; b: comparison between diabetic nontreated group (DC) and all groups; c: comparison between the diabetic group treated by olive oil (DC + OO) and the diabetic group treated by thyme honey (DC + TH); d: comparison between the diabetic group treated by the combination between olive oil and thyme honey (DC + OO + TH) and the groups treated by olive oil only (DC + OO) or thyme honey only (DC + TH).

**Table 9:** Effect of daily administration of thyme honey, olive oil, and their combination on pancreas enzymatic antioxidants of diabetic and nondiabetic rats.

Variables in the pancreas	Interventions						
	DW	OO	TH	DC	DC + OO	DC + TH	DC + OO + TH
Proteins (mg/g org)	7.23 ± 0.23^∗∗∗**b**^	7.20 ± 0.30^∗∗∗**b**^	7.18 ± 0.51^∗∗∗**b**^	2.75 ± 0.28^∗∗∗**a**^	4.83 ± 0.51^∗∗∗**a**∗∗∗**b**∗∗∗**c**∗∗∗**d**^	6.09 ± 0.19^∗∗∗**a**∗∗∗**b**∗∗**d**^	7.00 ± 0.21^∗∗∗**b**^
Catalase (*μ*mol H_2_O_2_/min/mg pr)	28.10 ± 2.73^∗∗∗**b**^	28.09 ± 1.96^∗∗∗**b**^	27.89 ± 1.99^∗∗∗**b**^	14.73 ± 1.07^∗∗∗**a**^	24.88 ± 1.09^∗∗∗**b**^	25.10 ± 1.72^∗∗∗**b**^	26.73 ± 2.91^∗∗∗**b**^
GSH (*μ*g/g org)	370.67 ± 3.55	370.85 ± 3.15	365.89 ± 4.87	108.67 ± 3.22	368.75 ± 5.23	323.52 ± 2.95	365.77 ± 3.91
GPx (nmol GSH/min/mg pr)	13.06 ± 0.61^∗∗∗**b**^	13.08 ± 0.66^∗∗∗**b**^	13.10 ± 0.85^∗∗∗**b**^	7.80 ± 0.56^∗∗∗**a**^	12.00 ± 1.06^∗∗∗**b**^	10.32 ± 0.94^∗∗∗**a**∗∗∗**b***∗ ***c***∗ ***d**^	12.06 ± 0.81^∗∗∗**b**^
MDA (nmol/g org)	26.32 ± 1.14^∗∗∗**b**^	26.40 ± 1.38^∗∗∗**b**^	25.96 ± 1.32^∗∗∗**b**^	54.80 ± 1.01^∗∗∗**a**^	36.31 ± 0.80^∗∗∗**a**∗∗∗**b**∗∗∗**c**∗∗∗**d**^	30.98 ± 0.50^∗∗∗**a**∗∗∗**b**^	32.09 ± 2.51^∗∗∗**a**∗∗∗**b**^

Data presented as mean ± SD; a: comparison between the distilled water group (DW) and all groups; b: comparison between the diabetic nontreated group (DC) and all groups; c: comparison between the diabetic group treated by olive oil (DC + OO) and the diabetic group treated by thyme honey (DC + TH); d: comparison between the diabetic group treated by the combination between olive oil and thyme honey (DC + OO + TH) and the groups treated by olive oil only (DC + OO) or thyme honey only (DC + TH).

## Data Availability

The data used to support the findings of this study are available from the corresponding author upon request.
